# Novel Tetra-Primer ARMS-PCR Assays for Thiopurine Intolerance Susceptibility Mutations *NUDT15* c.415C>T and *TPMT* c.719A>G (TPMT*3C) in East Asians

**DOI:** 10.3390/genes8100285

**Published:** 2017-10-23

**Authors:** Chi-Chun Ho, Wai-Ying Fong, Yuen-Hon Lee, Wing-Tat Poon

**Affiliations:** Department of Clinical Pathology, Pamela Youde Nethersole Eastern Hospital, Chai Wan, Hong Kong, China; hcc604@ha.org.hk (C.-C.H.); fwy669@ha.org.hk (W.-Y.F.); lyh101@ha.org.hk (Y.-H.L.)

**Keywords:** Asian, *NUDT15*, TPMT*3C, low-cost, genotyping, amplification-refractory mutation system, pharmacogenetic, rs1142345, rs116855232, sequencing, thiopurine intolerance

## Abstract

Thiopurines are clinically useful in the management of diverse immunological and malignant conditions. Nevertheless, these purine analogues can cause lethal myelosuppression, which may be prevented by prospective testing for variants in the thiopurine S-methyltransferase (*TPMT*) and, in East Asians, Nudix hydrolase 15 (*NUDT15*) genes. Two single-tube, tetra-primer amplification refractory mutation system polymerase chain reaction (ARMS-PCR) assays were developed to genotype the common loss-of-function variants *NUDT15* c.415C>T (rs116855232) and TPMT*3C c.719A>G (rs1142345). In a group of 60 unselected patients, one and seven were found to be homozygous and heterozygous, respectively, for *NUDT15* c.415C>T; one was found to be heterozygous for TPMT*3C c.719A>G. There was no non-specific amplification, and the genotypes were 100% concordant with Sanger sequencing. Limit-of-detection for both assays was below 1 ng of heterozygous template per reaction. Time- and cost-effective ARMS-PCR assays, suitable for genotyping East-Asian patients for thiopurine intolerance, were successfully developed and validated.

## 1. Introduction

Thiopurines are purine analogues with cytotoxic effect upon conversion to thioguanine nucleotides. Clinically, the thiopurines azathioprine (AZA), 6-mercaptopurine (6-MP) and 6-thioguanine (6-TG) are used in treatment of hematological malignancy (6-TG, 6-MP) [1,2,3,4], as steroid-sparing agents in ulcerative colitis and Crohn’s disease (AZA, 6-MP) [5,6,7,8] and, although now less commonly, as immunosuppressants in organ transplant recipients (AZA) [9]. Thiopurine treatment is limited by myelosuppression, which may lead to prematurely terminated or suboptimal treatment, and can complicate as severe neutropenia, sepsis and death [10]. The possible role of genetic variation in thiopurine metabolism and sensitivity has been noted since the 1980s [11], and testing of azathioprine intolerance due to non-functional thiopurine S-methyltransferase (*TPMT*) variants has been recommended by the US Food and Drug Administration since 2003 [12]. Prospective genotyping would allow the clinician to reduce thiopurine dosage for heterozygous patients, who may be unable to tolerate the full dose, and avoid administration of the drug in homozygotes, who are otherwise at fatal risk of profound myelosuppression. 

Non-functional *TPMT* alleles are common; the combined allele frequency (AF) of *3A (rs1800460, c.460G>A rs1142345, c.719A>G) and *3C (rs1142345, c.719A>G only) is as high as 8.1% and 10.9% in some Caucasian and African sub-populations [13]. In Asian populations, however, the overall prevalence of *TPMT* loss-of-function variants is lower (~1.7%) and is predominantly represented by the TPMT*3C variant (1.6%) [14]. Nevertheless, thiopurine-associated leukopenia in Asians is unexpectedly common, and often occurs in patients with a wildtype *TPMT* genotype [15]. In 2014, a Korean genetic association study uncovered the relationship between the common missense variant *NUDT15* c.415C>T (rs116855232) and susceptibility to thiopurine-induced leukopenia [16]. The allele is present in East Asians with AF greater than 10%, in South Asians and Latinos with AF 7%, but is only present in African and non-Finnish European populations with AF 0.07–0.4% [17]. In vitro, the active NUDT15 enzyme inactivates thiopurine metabolites and decreases its cytotoxicity, so patients with defective *NUDT15* alleles showed excessive thiopurine active metabolites and dose-dependent toxicity [18]. Based on these findings, up to nearly one-fifth of East-Asian patients may benefit from prospective genotyping in order to avoid significant morbidity [8,19,20] and mortality from severe unintended myelosuppression when thiopurine medications are prescribed [21]. Current clinical management guidelines recommend the determination of *TPMT* status, either by phenotypic testing of enzyme activity in circulating red blood cells, or by genotyping for known loss-of-function *TPMT* variants associated with enzyme deficiency [14,22,23]. International guidelines for *NUDT15* testing are still being developed [24].

Previously, our group compared the cost-effectiveness of direct sequencing, real-time PCR high-resolution melt (PCR-HRM) analysis and PCR-restriction fragment length polymorphism (PCR-RFLP) for genotyping of the variant alleles *NUDT15* c.415C>T and TPMT*3C and proposed PCR-HRM and PCR-RFLP as time- and cost-effective alternatives to Sanger sequencing [25]. To further streamline the workflow and reduce proprietary reagent costs, we developed two single-tube, tetra-primer amplification refractory mutation system polymerase chain reaction (ARMS-PCR) assays for genotyping the variants and validated our new assays against Sanger sequencing using 60 patient samples. Our experience confirmed that the ARMS-PCR assays developed are suitable for genotyping patients for potential thiopurine intolerance, in particular in East Asia, where the *NUDT15* c.415C>T variant is common, *TPMT* loss-of-function variants are dominated by the *3C allele, and the modest technical requirement of routine PCR is deemed cost-effective.

## 2. Materials and Methods 

### 2.1. Clinical Samples and DNA Extraction

Archived genomic DNA samples were retrieved from 60 patients who had been referred for genetic testing at our center. Patients presenting specifically for *NUDT15* or *TPMT* genetic testing were excluded from this study, as the current study represents part of an on-going study to determine local prevalence of the variant alleles. DNA extraction was performed as previously described [26]. Briefly, DNA from peripheral blood was extracted using the QIAamp DNA Blood Mini Kit (Qiagen, Hilden, Germany) following the manufacturer’s instructions and eluted in 100 μL of Tris-EDTA buffer. The extracted genomic DNA was stored at −80 °C until analysis. The patients gave written informed consent for anonymized testing and assay development using their samples. The study was approved by the Hong Kong Hospital Authority/Hong Kong East Cluster Institutional Review Board Ethics Committee (HKEC-2016-047; approval date: 23 August 2016).

### 2.2. ARMS-PCR Genotyping for NUDT15 c.415C>T and TPMT c.719A>G (TPMT*3C)

ARMS-PCR mix for *NUDT15* c.415C>T and *TPMT* c.719A>G (TPMT*3C) genotyping was prepared as follows: each 25 µL PCR reaction contained 10 ng purified genomic DNA, 12.5 µL AmpliTaq Gold 360 Master Mix (Applied Biosystems, Foster City, CA, USA), 2.0 µL of *NUDT15* genotyping primer mix or *TPMT* genotyping primer mix (Table 1), with the remaining volume added up to 25 µL by nuclease-free water (Thermo Fisher, Waltham, MA, USA). Thermocycling was performed on a Veriti 96-Well Thermal Cycler (Applied Biosystems) using a three-step PCR program as follows: initial denaturation at 95 °C for 10 min, followed by 35 cycles of denaturation at 95 °C for 1 min, annealing at 62 °C (for *NUDT15*) or 57 °C (for *TPMT*) for 45 s, extension at 72 °C for 45 s, and final extension at 72 °C for 10 min. Five microliters of PCR product from each reaction was electrophoresed in 2% agarose gel in 1 × TBE buffer at 100 V for 40 min, stained with GelStar (Lonza, Basel, Switzerland), and visualized under ultraviolet trans-illumination. To determine the analytical sensitivity of the assays, dilutions of template DNA from a sample with Sanger sequencing confirmed heterozygosity was made in nuclease-free water and added to the each of the *NUDT15* and *TPMT* genotyping ARMS-PCR reactions in final amounts of 10 ng, 5 ng, 2 ng, 1 ng, 0.5 ng, 0.2 ng and 0.1 ng. Experiments were performed in triplicate, and the lowest template amount at which visual inspection of product bands could still allow correct interpretation of the genotype in all three reactions was recorded as the lower limit of detection (LOD). To confirm the specificity of the PCR products, all visualized product bands from the LOD reaction were excised from the agarose gel, purified with the QIAquick Gel Extraction Kit (Qiagen) and sequenced using the respective primers as appropriate. 

### 2.3. Validation of Genotypes by Sanger Sequencing

Validation of genotypes of the clinical samples was performed as previously described [25]. Briefly, PCR amplification and Sanger sequencing for *NUDT15* was performed using the primers PCP-0023 (5′-CCCAAATAAACACCCTTTGTTTTCTGT-3′) and PCP-0024 (5′-CCTTTGTATCCCACCAGATGGTTC-3′), and for *TPMT* using primers PCP-0027 (5′-CACCCAGCCAATTTTGAGTA-3′) and PCP-0028 (5′-CAGGTAACACATGCTGATTGG-3′). PCR products were purified using EXO-SAP IT (Affymetrix, Santa Clara, CA, USA) according to the manufacturer’s protocol, dye-terminator labelled using the BigDye Terminator v1.1 Cycle Sequencing Kit and sequenced on an ABI 3500 genetic analyzer (Applied Biosystems). The sequence trace files obtained were compared with reference sequences NM_018283.3 (*NUDT15*) and NM_000367.4 (*TPMT*) using Mutation Surveyor version 4.0.9 (SoftGenetics, State College, PA, USA), and the determined genotypes were compared with the ARMS-PCR results.

## 3. Results

### 3.1. Genotypes of NUDT15 c.415C>T and TPMT*3C Variants by ARMS-PCR

**Figure 1:**
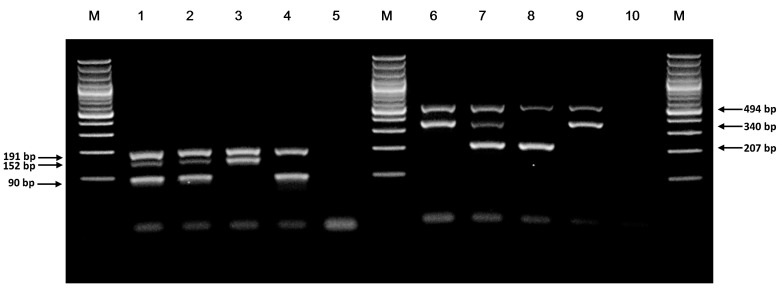
Genotyping of *NUDT15* c.415C>T and TPMT*3C variants by ARMS-PCR. Lane M, GeneRuler 100 bp DNA Ladder (#SM0241, Thermo Fisher); lane 1, anonymized patient sample 17C-5685946 for *NUDT15* c.415C>T genotyping (compatible with heterozygous genotype); lane 2, *NUDT15* ARMS-PCR heterozygous (C/T) sample; lane 3, *NUDT15* ARMS-PCR homozygous mutant (T/T) sample; lane 4, *NUDT15* ARMS-PCR homozygous wildtype (C/C) sample; lane 5, *NUDT15* ARMS-PCR no DNA control; lane 6, sample for TPMT*3C from same patient as lane 1 (compatible with homozygous wildtype genotype); lane 7, *TPMT* ARMS-PCR heterozygous (A/G) sample; lane 8, *TPMT* ARMS-PCR synthetic DNA fragment for G allele (G/G); lane 9, *TPMT* ARMS-PCR homozygous wildtype (A/A) sample; lane 10, *TPMT* ARMS-PCR no DNA control.

From in silico prediction, for the *NUDT15* c.415C>T genotyping reaction, three product bands of size 191 bp (common amplicon), 152 bp (mutant/T-allele-specific amplicon) and 90 bp (wildtype/C allele-specific amplicon) were expected for heterozygotes; two bands of 191 bp and 90 bp were expected for wildtype homozygotes; and two bands of 191 bp and 152 bp were expected for mutant homozygotes. Out of 60 patient samples, 52 (86.7%) had two bands of size 191 bp and 90 bp, corresponding to a wildtype homozygote status; seven (11.7%) had all three bands, corresponding to a heterozygote status; and one (1.7%) had two bands of size 191 bp and 152 bp, corresponding to a homozygous mutant (T/T) status (Figure 1).

The product bands predicted for the TPMT*3C (c.719A>G) genotyping ARMS-PCR were: 494 bp (common amplicon), 340 bp (wildtype/A allele-specific amplicon) and 207 bp (mutant/G allele-specific amplicon). Fifty-nine patients (98.3%) had two bands of size 494 bp and 340 bp, corresponding to a wildtype homozygote (A/A) status; one patient (1.7%) had all three bands, corresponding to a heterozygote (A/G) status (Figure 1). 

### 3.2. Comparison of ARMS-PCR with Conventional Sanger Sequencing

By Sanger sequencing and software-assisted sequence comparison, 52 (86.7%), 7 (11.7%) and 1 (1.7%) patients were found to be homozygous for the wildtype allele (C/C), heterozygous (C/T) and homozygous for the mutant allele (T/T), respectively, for the *NUDT15* c.415C>T variant site. For the TPMT*3C (c.719A>G) variant site, 59 patients (98.3%) and 1 patient (1.7%) were found to be homozygous for the wildtype allele (A/A) and heterozygous (A/G), respectively. None of the patients harbored both *NUDT15* c.415C>T and TPMT*3C variants. All ARMS-PCR results were concordant with the conventional Sanger sequencing findings. 

### 3.3. Sensitivity and Specificity of ARMS-PCR Assay

**Figure 2:**
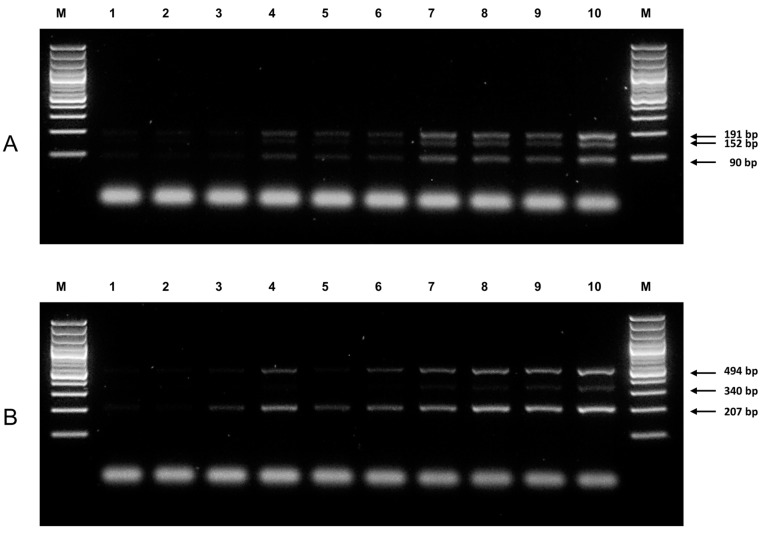
Limit of detection (LOD) determination for *NUDT15* and *TPMT* genotyping reactions. Lane M, GeneRuler 100 bp DNA Ladder (#SM0241, Thermo Fisher); lanes 1–3, 0.1 ng template DNA; lanes 4–6, 0.2 ng template DNA; lanes 7–9, 0.5 ng template DNA; lane 10, 1 ng template DNA. (**A**) Visual detection of all three bands for a sample heterozygous for *NUDT15* c.415C>T variant was possible down to 0.5 ng of template DNA per 25 µL reaction; (**B**) Visual detection of all three bands for a sample heterozygous for TPMT*3C variant was possible down to 0.5 ng of template DNA per 25 µL reaction.

As heterozygous samples contain approximately half the copies of each of the specific alleles as templates, and that the presence of both non-refractory (or “amplifiable”) templates would theoretically lead to more competition for DNA polymerase and free nucleotides, sensitivity of the ARMS-PCR assays developed was tested using heterozygous samples from each locus. For the *NUDT15* c.415C>T genotyping reaction, the LOD was determined to be 0.5 ng of genomic DNA per 25 µL of reaction (Figure 2A); for TPMT*3C genotyping, the LOD was determined to be 0.5 ng (Figure 2B). For all genotyping reactions, no non-specific amplification of the alternate allele was observed for the homozygous samples. DNA sequencing confirmed the identity of all the amplicons. 

## 4. Discussion

ARMS-PCR is a time-efficient technique which combines the amplification and genotyping steps into a single reaction [27]. The tetra-primer configuration employed in this study includes a co-amplified common amplicon as internal control [28] and, unlike other “one-step” techniques, such as high-resolution melt analysis, is relatively robust to minor variation in template DNA concentration. Moreover, as only PCR reagents and standard oligonucleotide primers are needed, such assays are particularly suited to molecular laboratories which may find the cost and shelf-life of special fluorescent dyes and special restriction enzymes prohibitive [29,30,31]. As such, tetra-primer ARMS-PCR has been adopted and advocated by clinical laboratories in genotyping clinically important single-nucleotide polymorphisms [32]. While multiplexed allele-specific PCR for the detection of common *TPMT* variants have been previously published, such assays required two PCR reactions per patient sample to discriminate between homozygotes and heterozygotes [33,34], or did not cover the *3C variant [32]; additionally, the internal control amplicon of the some of the assays targeted a different genomic region distant from the *TPMT* gene [33], which, theoretically, could give falsely-reassuring results when there is gross deletion of the targeted pharmacogenetic locus [35]. 

In this study, we extended our previous work on *NUDT15* c.415C>T and TPMT*3C genotyping and devised two highly time- and cost-efficient ARMS-PCR assays. Compared to the methods previously described—namely, Sanger sequencing, PCR-HRM and PCR-RFLP—the current method tolerates the lowest amount of input DNA, has the lowest cost per sample and has a straightforward interpretation [25]. Although PCR-RFLP also has simple interpretation and minimal requirement for molecular equipment, misinterpretation of *TPMT* genotypes due to incomplete restriction endonuclease digestion has been reported [36]. Compared to our reagent cost of Sanger sequencing genotyping (~USD 7.0), which included amplicon preparation, sequencing reaction setup and post-sequencing cleanup, the reagent cost of the ARMS-PCR assay was about 50% lower (~USD 3.6) (Table S1). For smaller clinical laboratories that may not have their own capillary sequencer, the ARMS-PCR genotyping protocol also provides a means for them to provide thiopurine pharmacogenetic testing as an in-house assay, obviating the need to procure and maintain their own Sanger sequencing equipment [37]. In fact, since the successful development of the ARMS-PCR assays, we have phased-out the routine use of Sanger sequencing and software-assisted mutational analysis for genotyping the two thiopurine-intolerance susceptibility loci for East Asian patients, and reserved the sequencing test for patients of non-East-Asian ethnic origin. Nevertheless, we note that Sanger sequencing cannot currently be replaced in the clinical laboratory, as it remains the method of choice to delineate the full range of single nucleotide polymorphisms and small indels in routine clinical samples, and serves as a confirmatory and validation tool if there exists discrepancy amongst alternative assays or when the targeted methods fail to yield expected results [38].

Limitations of this study included the lack of validation of the TPMT*3C assay against homozygous mutant (G/G) patient samples, due to its rarity in our population; and that the band intensity of the control and allele-specific bands could not be completely equalized, despite extensive optimizations. However, as pointed out in a methodological review [39], neither normally affects the interpretation and validity of the results. 

## Figures and Tables

**Table 1:** Primers used for amplification refractory mutation system polymerase chain reaction (ARMS-PCR) genotyping.

Primer	Sequence	T_m_ (°C) ^1^	Expected Product Size (bp)	Final Concentration (µM) ^2^
*NUDT15* c.415C>T genotyping				
N-OF	5′-CCCAAATAAACACCCTTTGTTTTCTGT-3′	55.2	191	0.18
N-OR	5′-CCTTTGTATCCCACCAGATGGTTC-3′	57.4	191	0.18
N-WT	5′-GGACCAGCTTTTCTGGGGACTAC-3′	58.8	90	0.82
N-MT	5′-GGATCATAGCCTTGTTCTTTTAAACAATA-3′	54.4	152	0.82
*TPMT* c.719A>G (TPMT*3C) genotyping				
T-OF	5′-CACCCAGCCAATTTTGAGTA-3′	49.7	494	0.1
T-OR	5′-CAGGTAACACATGCTGATTGG-3′	52.4	494	0.1
T-MT	5′-ATGTCTCATTTACTTTTCTGTAAGTACAC-3′	54.4	207	0.9
T-WT	5′-TTGACTGTCTTTTTGAAAAGTTCTA-3′	49.5	340	0.9

^1^ Melting temperatures (Tm) estimated using the OligoCalc (http://biotools.nubic.northwestern.edu/OligoCalc.html) using the algorithm for basic Tm calculation; ^2^ Stock solution of each primer was first diluted to 25 µM with 1× Tris-EDTA buffer. The 25 µM working primer solutions for *NUDT15* genotyping were then combined in volume ratios 2:2:9:9, and that for *TPMT* genotyping in ratios 1:1:9:9. Two microliters of the tetra-primer mix (“primer mix”) was used in each 25 µL ARMS-PCR reaction. *NUDT*: Nudix hydrolase 15 gene; *TPMT*: thiopurine S-methyltransferase gene; OF: outer forward; OR: outer reverse; WT: wildtype; MT: mutant.

## Supplementary Materials

The following are available online at www.mdpi.com/2073-4425/8/10/285/s1. Table S1: Reagent cost of Sanger sequencing and ARMS-PCR in the clinical laboratory.
