# Supplementary material for: Novel Tetra-Primer ARMS-PCR Assays for Thiopurine Intolerance Susceptibility Mutations NUDT15 c.415C>T and TPMT c.719A>G (TPMT*3C) in East Asians
Source: Genes (Basel). 2017 Oct 23;8(10):285. doi: 10.3390/genes8100285 (PMC5664135; doi:10.3390/genes8100285)
Supplement: Supplementary file 1 [file genes-08-00285-s001.docx]

**Table S1. Reagent cost of Sanger sequencing and ARMS-PCR in the clinical laboratory**

The following cost-calculations used prices quoted from official websites / publicly available price lists where applicable. Substitutes have been made where reagents were available to our laboratory at special discounts, in order to better reflect the true reagent cost. All prices were based on respective local pricing where our laboratory is based (Hong Kong, China), unless otherwise stated. The cost of laboratory plasticware (e.g. pipette tips, PCR tubes), equipment (thermocycler, Sanger sequencing instrument) and maintenance fee was excluded from the calculation. All costs converted using HKD 7.8 = USD 1.

|  |  | ***Sanger sequencing reagent cost (per 25 µL)*** | ***Tetra-primer ARMS-PCR***  ***reagent cost (per 25 µL)*** | ***Unit price*** | ***Source*** |
| --- | --- | --- | --- | --- | --- |
| *Amplicon preparation* | Polymerase master mix | USD 0.71 | USD 0.71 | USD 0.056 / µL  (12.5 µL of 2× mix per reaction) | https://www.thermofisher.com/order/catalog/product/4398881 |
|  | Nuclease-free water | USD 0.017 | USD 0.017 | USD 0.0013 / µL  (approx. 12.5 µL per reaction) | https://www.thermofisher.com/order/catalog/product/R0582?SID=srch-srp-R0582 |
|  | Primers | USD 0.016 | USD 0.032 | USD 8.1/ 0.025 µmole of 30-bp custom oligo  (assume final concentration of each primer = 1.0 µM) | http://cgs.hku.hk/portal/index.php/oligo-service/service-charges (commercial pricing) |

|  |  | ***Sanger sequencing reagent cost (per 25 µL)*** | ***Tetra-primer ARMS-PCR***  ***reagent cost (per 25 µL)*** | ***Unit price*** | ***Source*** |
| --- | --- | --- | --- | --- | --- |
| *Analytical gel electrophoresis* | Agarose gel | skipped* | USD 0.6 | USD 1.2 / g  (assume gel volume 50 mL per slab of 2% agarose gel; half gel used) | https://www.thermofisher.com/order/catalog/product/16500500?SID=srch-srp-16500500 |
|  | Electrophoresis buffer | skipped* | USD 0.98 | USD 0.98 / 250 mL of 1× buffer (assuming fresh buffer used every time) | https://www.thermofisher.com/order/catalog/product/15581044?SID=srch-srp-15581044 |
|  | Nucleic acid gel stain | skipped* | USD 1.3 | USD 0.51 / µL (assume 5 µL added to each gel; half gel used) | https://www.fishersci.com/shop/products/lonza-gelstar-nucleic-acid-gel-stain-2-250-l/bma50535 [US pricing used] |
| *PCR product cleanup* | Exonuclease and shrimp alkaline phorphatase mix | USD 1.2 | N.A. | USD 1.2 / reaction | https://www.fishersci.com/shop/products/exosap-it-express-pcr-100react/ 501128619 [US pricing used] |
| *Dye terminator sequencing* | Dye terminator sequencing mix | USD 2.5 | N.A. | USD 2.5 / reaction (assume ¼ reaction used for cost-savings) | https://www.thermofisher.com/order/catalog/product/4337450?SID=srch-srp-4337450 [local quote used] |
|  |  |  |  |  |  |
|  | Post-sequencing purification | USD 2.6 | N.A. | USD 2.6 / reaction | https://www.thermofisher.com/order/catalog/product/4376486 [local quote used] |
| ***Total reagent cost*** |  | ***USD 7.0*** | ***USD 3.6*** |  |  |

* Assuming in highly optimized protocols the pre-sequencing visualization step may be skipped.
